# ASAS–NANP symposium: Mathematical modeling in animal nutrition: Integrating insight and uncertainty: Bayesian modeling for data-rich livestock systems

**DOI:** 10.1093/jas/skag307

**Published:** 2026-09-29

**Authors:** Ira L Parsons, Hector M Menendez, Gordon E Carstens, Jameson R Brennan

**Affiliations:** West River Research and Extension Center, Department of Animal Science, South Dakota State University, Rapid City, SD 57703, United States; West River Research and Extension Center, Department of Animal Science, South Dakota State University, Rapid City, SD 57703, United States; Department of Animal Science, Texas A&M University, College Station, TX 77845, United States; West River Research and Extension Center, Department of Animal Science, South Dakota State University, Rapid City, SD 57703, United States

**Keywords:** animal science, bayesian inference, beef cattle, precision livestock technologies, dry matter intake, Markov chain Monte Carlo (MCMC)

## Abstract

Bayesian statistical modeling provides a principled framework for integrating biological knowledge, process understanding, and observed data in animal science research. By treating all unobserved quantities as random variables and explicitly defining probability distributions for both processes and observations, Bayesian methods allow researchers to quantify uncertainty, incorporate prior information, and evaluate full distributions of plausible outcomes. This manuscript introduces the foundational concepts of Bayes’ theorem, probability, probability distributions, and Markov chain Monte Carlo, and demonstrates how these components form coherent, process-based statistical models. Two case studies illustrate the application of Bayesian regression to questions central to beef cattle research: the influence of day-to-day variation in dry matter intake on average daily gain, and the prediction of intake from biometric variables collected using precision livestock technologies. Together, these examples highlight how Bayesian approaches enhance model transparency, improve interpretation, and support decision-making in complex biological systems. This framework equips animal scientists with the tools needed to ask clearer questions, build biologically grounded models, and leverage emerging data streams to advance research and management. Keywords: animal science, bayesian inference, beef cattle, precision livestock technologies, dry matter intake, Markov chain Monte Carlo (MCMC).

## Teaser

Bayesian modeling gives animal scientists a flexible, transparent means to develop and test biologically meaningful models with real data and quantify uncertainty in intuitively meaningful ways. This manuscript illustrates how linking prior knowledge with specific research objectives, using probabilistic outcomes, and leveraging modern computational power can inform updated knowledge and build stronger, more informative models that elevate research questions.

## Introduction

Bayes’ theorem was first proposed by Thomas Bayes, an English statistician, philosopher, and minister in the 1700s. Bayes famously never published the theory that bears his name, but the theorem and proofs were found and later published by his friend Richard Price. Working from his role as a minister, Bayes was motivated to develop his theory based on a desire to understand the world and provide mathematical formulas surrounding origins of the universe and the existence of a creator or other beginning. His theorems have been interpreted in many ways by multiple people but can be summarized as an interest in the relative strength of beliefs and hypotheses rather than the frequency of their being true. His work was popularized by the French scholar Pierre-Simon Laplace, who brought statistical probability to Bayes’ interpretation of probability, popularizing the term in the late 19th century of LaPlaces’ theory. Today, statisticians who view the world in probabilistic terms based upon prior knowledge of the system refer to themselves as “Bayesians” and prefer to measure the probabilistic strength of a hypothesis rather than strictly accepting or rejecting a hypothesis as is practiced by frequentists using Null Hypothesis Testing. The methodology has garnered varying degrees of acceptance in the sciences, ranging from almost cult-like following amongst its adherents to outright scorn by other frequentist statisticians of the period. Fischer, arguably one of the most influential frequentist statisticians of the twentieth century, famously summarized probabilistic statistics with the phrase “to be a Bayesian is to never have to admit you are wrong.”

It is useful to briefly describe the three traditional frameworks for evaluating statistical significance. The most common, at least in basic statistics courses, is frequentist statistics, which assumes there is a true state of the world and seeks to calculate the long-run probability of a particular outcome based upon a sequence of observations arising from repeated experiments (Bolker 2008). Maximum likelihood estimation is the fundamental core of most modern scientific methods and identifies the set of parameters that would most likely have occurred given a set of data, and it shares the same philosophical underpinnings as frequentist statistics. On the other hand, Bayesian philosophy takes a mirror-image to this approach, which holds that the experimental outcome represents the true state of affairs, while the parameter values in our model are randomly drawn from a distribution of possible values (Bolker 2008; Hobbs and Hooten 2015; Hooten and Hefley 2019). Most notably, the Bayesian approach offer unique advantages for updating your prior beliefs in the context of more information, which makes it incredibly useful when approaching challenges such as the diagnostic tests.

## Materials and Methods

### Models

Utilizing theoretical and systems models to describe expected scientific hypotheses or systems-level outcomes should be familiar. Models represent simplified abstractions regarding the operation of a system, and purposefully omit details or variables to provide insight into the system function (Brennan et al. 2023). Conceptual models aid in developing research hypothesis and experimental designs by providing a mathematical framework that relates variables to one another. Statistics provide the scientific uncertainty regarding the confidence we have in the conclusions based on the evidence (Hobbs and Hooten 2015).

Even though we know that “all models are wrong, but some are useful,” conceptual and mathematical models are integral to understanding complex ecological and biological systems (Hyde et al. 2022; Aruna et al. 2024; Menendez et al. 2025). By providing a framework for research processes, they guide scientists to gain understanding by asking questions that directly relate to the system, while identifying both areas where knowledge is plentiful and problems yet to be addressed (Bolker 2008). Biological systems are inherently complex leading researchers to take a multiple hypothesis testing approach to analyzing scientific data, which allows them to build a body of inference in support of one hypothesis (Chamberlin 1965; Rehme et al. 2011). This method allows researchers to consider and challenge prevailing hypothesis (Chamberlin 1965) and works well with the Bayesian approach, which facilitates considering a range of potential outcomes given the variation expected in the system. The modeling process closely follows the scientific method and serves as a useful outline for both developing, conducting, and communicating scientific findings (Ott and Longnecker 2016).

Upon identifying a problem, the researcher should first define the question they wish to answer at both general and specific levels (Figure 1). For example, the question may generally be, “Does yeast inclusion in heifer development diets improve dry matter intake in weaned heifers?,” and specifically be “What is the expected average difference in dry matter intake measured in Kgs of heifers fed yeast vs. those not?” This first step involves developing a theoretical model that includes the basic components, or variables, we theorize are important to the system we are studying (Hobbs and Hooten 2015). A critical component of developing quality research and communicating research findings is learning to intentionally and clearly move between these two levels during the modeling and communication processes (Bolker 2008). After identifying and carefully defining the question, the second step is to develop a deterministic or empirical model that captures our understanding of the process under study. This allows the mathematical expression of our mental conceptual model, and facilitates the prediction of outcomes we can compare observed data to (Hobbs and Hooten 2015).


(1) DMI=β0+β1(Yeast1,2),

**Figure 1:**
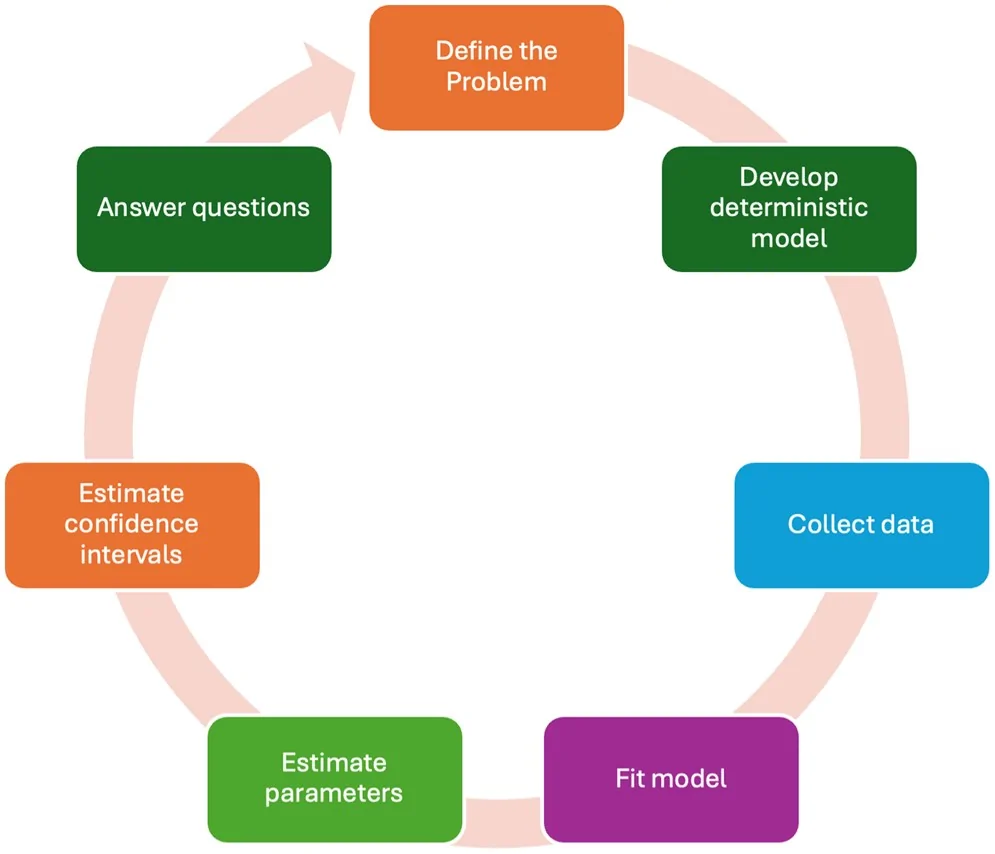
The scientific process closely aligns with model development, providing a succinct roadmap for researchers to follow as they develop models, ask scientific questions, fit data to models, derive statistical results, and communicate scientific findings. Adapted from Ott and Longnecker (2016).

where DMI is the daily dry matter intake, β_0_ is the *y* intercept, and β_1_ is the slope of the regression line of DMI on yeast inclusion, which is listed as (Yeast_1,2_), indicating either yeast inclusion or not in the heifer development ration. A close study and simulation of the empirical model aids in the development of robust experimental designs that allow the isolation of key variables of interest and the planning of carefully constructed studies to be employed in the third step of collecting observational data from the system of interest (Figure 1). Following our heifer development example, this involves developing an experiment where we feed heifers a development ration formulated to include yeast or not, and then observe their dry matter intake, perhaps using a precision livestock technology such as a feed intake bunk system, which assigns feed consumption to individual animals.

Fitting the empirical model to the collected data requires accounting for variation within the data. Choosing the stochastic model is the fourth step in the modeling process and enables both parameter estimation and the description of the probability distributions used to quantify the uncertainty in the observed variables. This technical step is one that is often overlooked, but the philosophy with which we approach; it is the largest defining step that separates Bayesian from traditional, or frequentist, approaches to model fitting. The fifth step of parameter estimation. Finally, we approach the sixth step, where we can assess the results of our research, utilize the models to aid in communicating our findings, and formulate new scientific hypotheses informed by our discoveries.

### Bayes’ theorem

Inverse probability theory divides the world into things that are observed and things which are unobserved. This works into Bayes’ theorem which translates to the mathematical formula used to update probabilities based upon the acquisition and observation of new evidence.


(2) $$P(\theta |y)=\frac{P(y|\theta )\;*\;P(\theta )}{P(y)},$$

where P(θ|y): posterior probability of event θ given evidence of event y, P(y|θ): likelihood of evidence θ given event y, P(θ): prior probability of event θ, and P(y): probability of evidence y (normalizing constant).

In essence, this construction allows the revision of the probability hypothesis (θ) given new evidence (y) through combining prior beliefs with the likelihood of observing the evidence. This construction is widely used in statistics, particularly in the fields of machine learning and decision-making tasks like spam filtering, medical diagnosis etc. Uniquely, all unobserved quantities are incorporated into the model as random variables, which are governed by chance according to the underlying support structure of the probability distribution. The support structure essentially defines the probability distribution that governs the data type and the space where the probability of any given random variable occurs (Hobbs and Hooten 2015).

### Probability

What makes Bayesian statistics unique is the fact that it treats all unobserved quantities as random (Hobbs and Hooten 2015). To apply this to datasets requires understanding the basics principles of probability and probability distributions. Consider a sample space represented by S which represents all the possible outcomes which might be possible from a given variable in an experiment (Figure 2). Suppose that we draw a sample from sample space S, which gives us the values of A. The probability of A occurring can be quantified as


(3) P(A)=Area of AArea of S.

**Figure 2:**
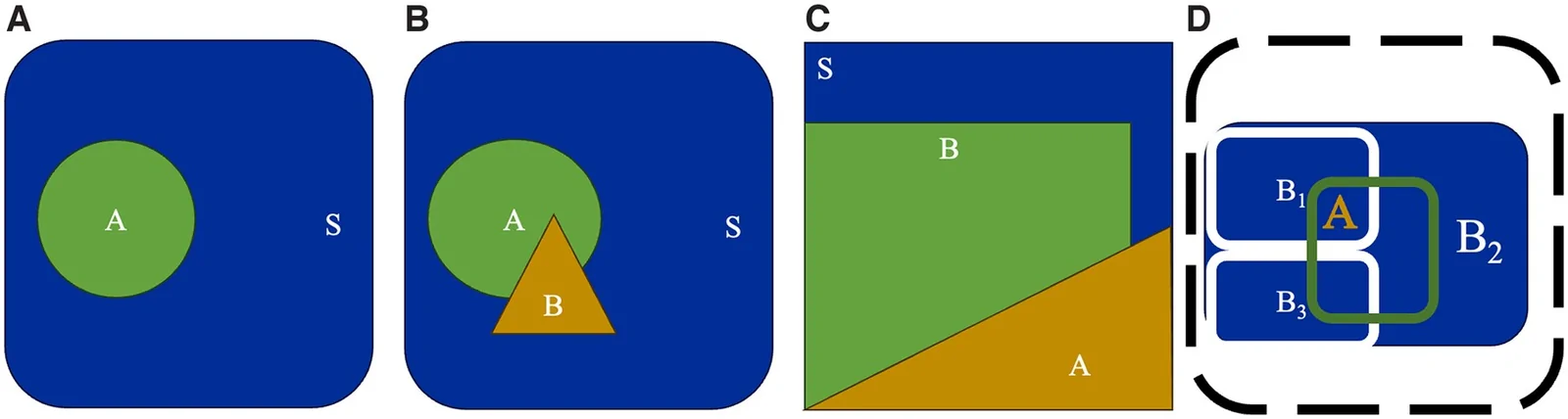
Types of conditional probability, starting with A) Proportional, B) Conditional, C) Independent, and D) Total Probability. inspired by Hobbs and Hooten, (2015).

Introducing a second variable B which is related to A in some way allows us to quantify the probability of observing B within the context of P(A), such that the


(4) P(B|A)=P(A,B)P(A).

A more thorough understanding of probability is necessary to completely appreciate fully how to formulate Bayesian networks. However, fully covering that subject is outside the fundamental scope of this paper, and readers are encouraged to pursue information in Hobbs and Hooten (2015) for more information. However, it is suffice to understand that random variables are governed by probability distributions which may be factored based upon the context of the space they occupy and overlapping space.

Graphically, this is illustrated through Bayesian Networks, also known as Directed Acyclic Graphs (DAG) or DAGs (Figure 3). Bayesian networks graphically specify how the joint distributions are factored into conditional distributions, where nodes are used to represent random variables, and arrows describe conditioning. As a rule, nodes at the heads of arrows must be variables written to the left of factored distributions, and nodes at the tails are written to the right. Remember that one of the unique powers of using the Bayesian approach to statistical modeling is the freedom to develop your own personal model based upon your expertise in the field. A DAG is useful in this regard, and using an eraser board, is your best friend as you define your variables and orient them in a manner that uniquely answers the scientific questions you have posed. Those who are familiar with systems dynamic modeling, popularized by Sterman (2010) and extensively applied to animal science (Tedeschi et al. 2005; Turner et al. 2016), will recognize the utility in this approach. However, it is important to note that the Bayesian network does not include feedback loops to update the distributions at the tails, as this would break the model.

**Figure 3:**
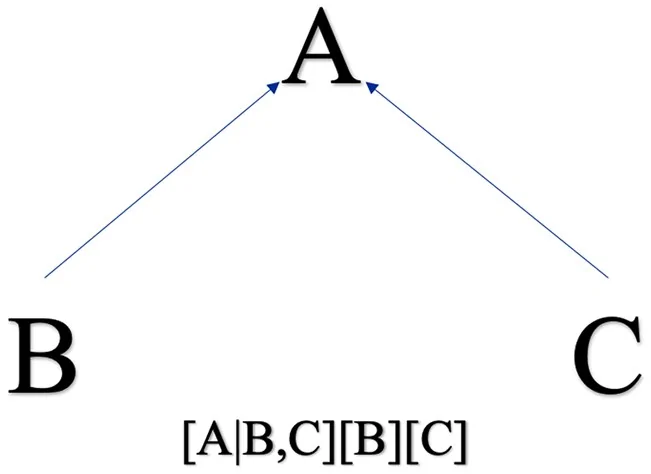
Demonstration of how a conditional distribution is factored into conditional distributions using a Bayesian Network. Brackets indicate a random variable with a probability distribution.

#### Probability distributions

Defining something as a probability indicates a probability distribution. As we are all intuitively aware, some outcomes are more probable than others. For example, the average height of men in America takes on a normal distribution with a typical male in America is 5′9″ (175 cm) with a standard deviation (SD) of 3″ (7.1 cm). Men who are 6′ (183 cm) tall represent the 85th percentile, meaning that 183 cm is taller than 85% of American men, and only 15% (100–85%) of men are taller. Suppose that we are meeting a new person whom we know to be a man, a statistician would conclude that there is a 15% chance that he is taller than 183 cm, and the chance that he is 7′ (213 cm) tall is not zero, but less than 0.000019%, or in practical terms, extremely unlikely.

Probability distributions represent the uncertainty we have around variables by defining the space we expect them to take up and represent our toolbox for fitting models to our data. Probability distributions are defined by their “moments,” which we use to describe both their central tendency point of data that is the most likely or common, and the range of values and their likelihood of occurring. Probability distributions are supported by their underlying data, with the data type defining what type of distribution we might choose. Refer to Table 1 for common probability distributions, the data they can be supported by along with their descriptive parameters and moments.

**Table 1:** Common probability distributions.

Probability model	Random variable support	Parameters	Moments
**Normal**	Real numbers	u, σ2	u, σ2
**Lognormal**	Positive real numbers	α mean of log of z β the standard deviation of the log of z	u, σ2
**Gamma**	Positive real numbers	α = shape, β = rate	u, σ2
**Beta**	Real numbers [0,1] or (0,1)	α, β	
**Bernoulli**	0 or 1	ɸ probability that random variable equals 1 ɸ = u	u = ɸ σ2 = ɸ(1-ɸ)
**Binomial**	Counts in two categories with upper bound	*n* number of trials ɸ probability of success	u = nɸ σ2 = nɸ(1-ɸ)
**Negative binomial**	Counts	λ the mean number of occurrences k dispersion parameter	u = λ Σ2 = λ + λ2/k

### Markov chain Monte Carlo

As we alluded to in our discussion on Bayes theorem, the probability density of a variable is calculated by dividing the joint distribution by the marginal distribution to create a probability density function (Yazdi et al. 1999; Hobbs and Hooten 2015). This probability density function is what allows Bayesian models to treat all unknown variables as random values that arise from an unknown probability distribution. Combining distributions is accomplished through a process of conjugation, also known as factoring the joint probability or calculating the marginal distribution. If we are lucky and the distributions supported by our prior and data share common moments (Table 1), this can be accomplished mathematically using calculus. For example, the conjugated distribution between a Normal and Lognormal or Beta distribution can easily be calculated by multiplying them together and dividing over a normalizing constant using matrix algebra because the share common moments of *u* (mu) and σ (rho). However, this is often not the case, and we might need to conjugate a normal distribution with a binomial distribution, which has the moments of *u* and ɸ (phi). This also becomes more difficult and rises to the level of impossible as the number of variables increase (Hobbs and Hooten 2015). This requires a different strategy that is solved by brute computational force using Markov chain Monte Carlo sampling to assess the shape of the joint distribution (Gelfand et al. 1990; Gelfand and Smith 1990). Markov chain Monte Carlo sampling normalizes the conjugate distributions by repeatedly sampling the posterior distribution and accumulating them into a stored vector, known as a chain. By repeatedly sampling the posterior distribution, we can infer its true shape, while allowing it to be normalized such that the area under the density curve sums to 1. This allows us to use the distributions to calculate the probability of observing a particular value, as we saw in the example for observing a man over 7′ (213 cm) tall.

## Results

### Case studies and applications

In the following sections, we present two case studies using Bayesian methods to analyze the study. Analysis was conducted in version 4.5.2 of Program R (R Core Team 2025), using packages *data.table* (Barrett et al. 2006) for data wrangling, *tidyverse* (Wickham et al. 2019) for plotting and summary statistics, and *rjags* (Martyn Plummer 2025) to fit Bayesian models, and *MCMCvis* (Youngflesh 2018).

### Case study 1: influence of day-to-day variation of dry matter intake on average daily gain

It has been noted that cattle in feed efficiency tests with greater day-to-day variation in dry matter intake exhibit lower average daily gains and higher residual feed intake (Parsons et al. 2020). This leads us to our research hypothesis “Animals with demonstrated decrease in day-to-day variation in feed intake will demonstrate improved metrics for performance and feed efficiency.” We will use example data collected in a feeding study (Figures 4 and 5) conducted with weaned heifers fed in a precision feed intake system (Growsafe, Vytelle). Code and example analysis are available in the online workbook presented at the 2025 NANP symposium held at the ASAS national meetings in Hollywood, FL (Parsons 2025; Parsons and Carstens 2026). The sample dataset has been cleaned to remove failed intake days and summarized to total daily intake using methods described in Parsons et al. (2020). Animals were weighed weekly using a livestock chute to obtain full bodyweight, which was used to calculate average daily gain via linear regression.

**Figure 4:**
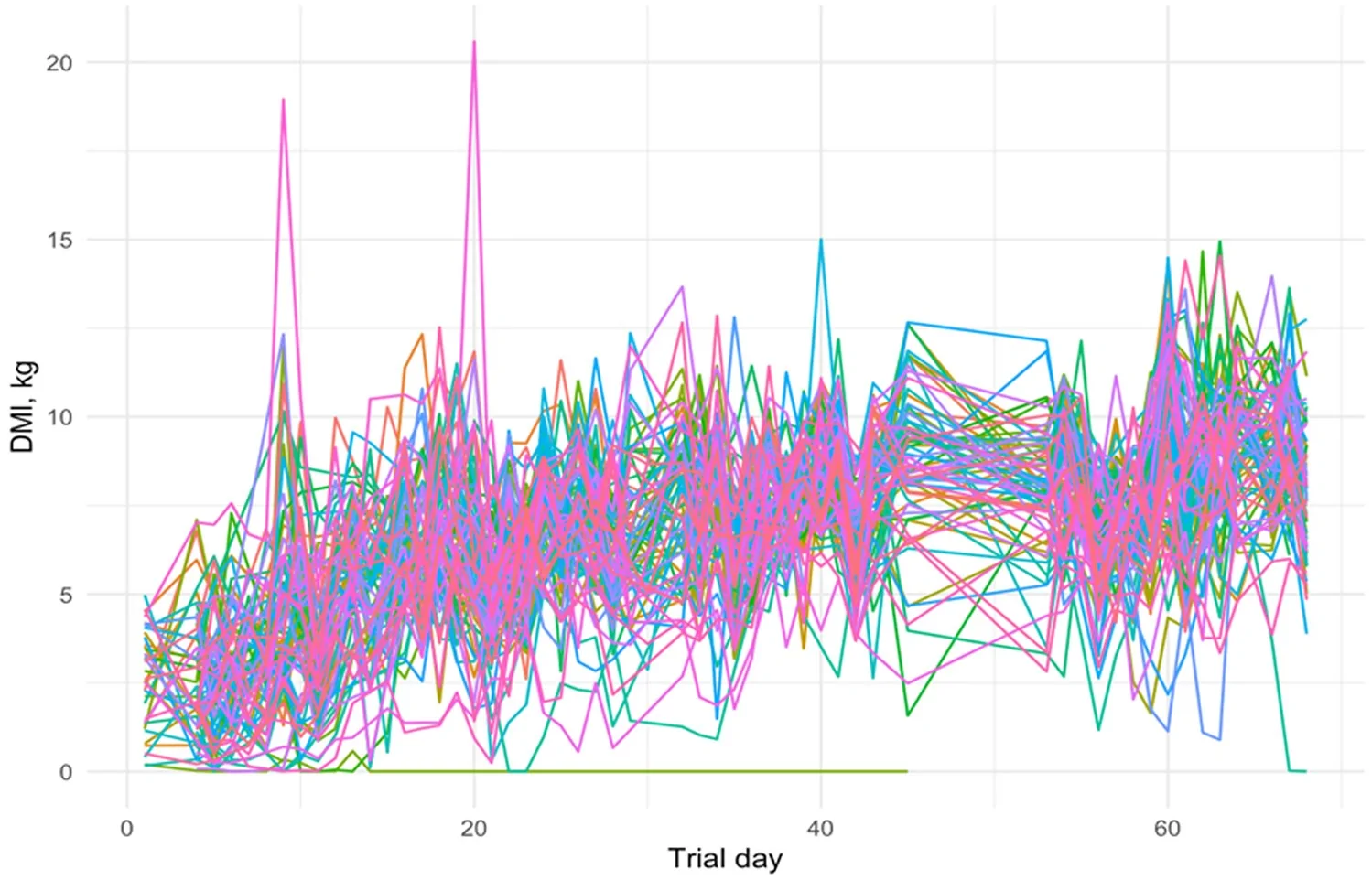
Daily feed intake across the feeding period.

**Figure 5:**
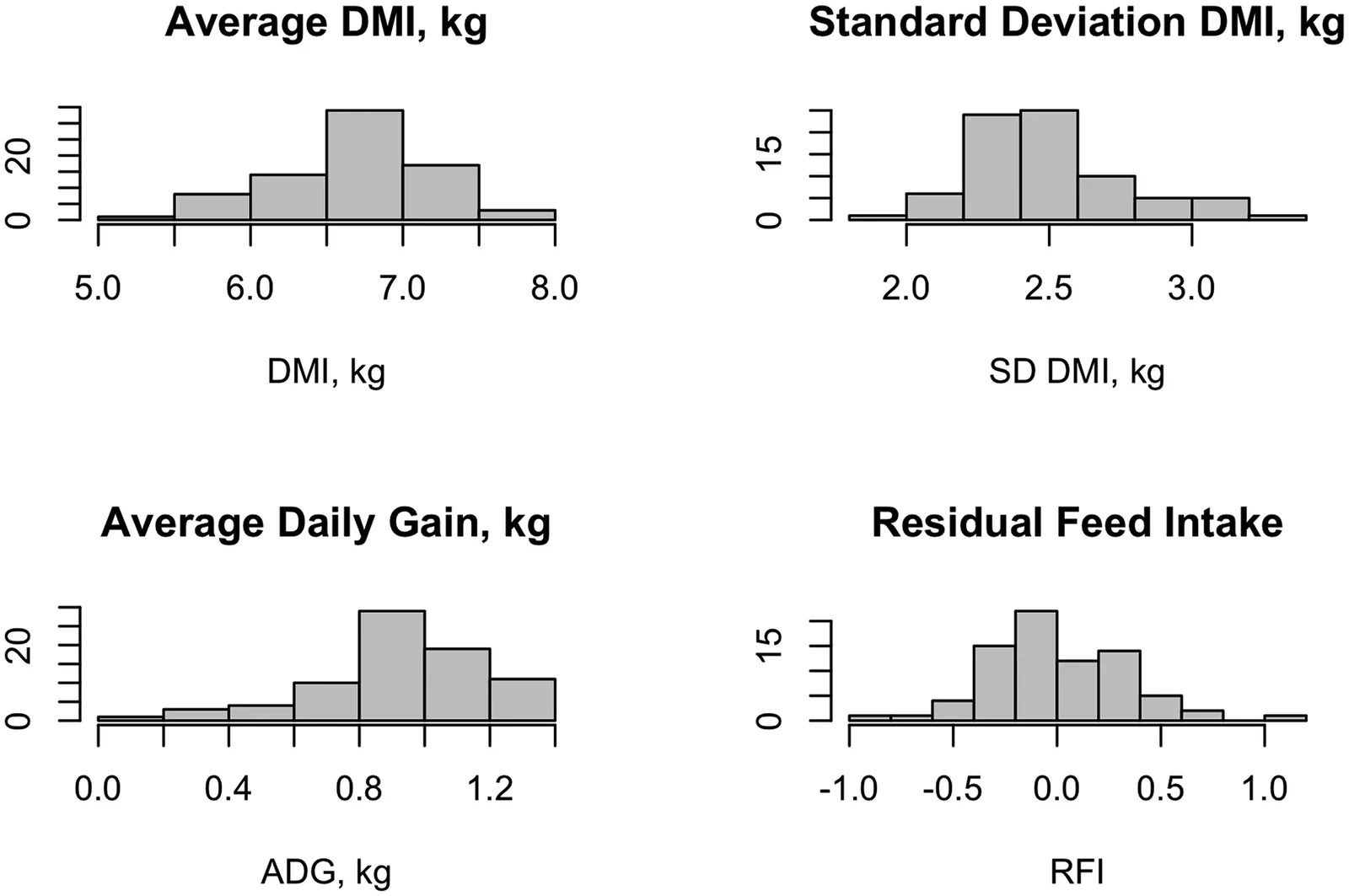
Summary statistics for each of the variables we will use to test our hypothesis and satisfy the research objectives using Bayesian approach to statistical modeling.

#### Case study 1—objectives and model

After identifying our dataset, which we can use to test our hypothesis, it is a crucial step to clearly define our research objective. In this case, our research objective is to “Utilize Bayesian regression methods to test the effect of increased variation in day-to-day dry matter intake on individual average daily gain.” We chose a linear regression model with the form


(5) g(β, xi)=N(β0+ β1xi+σ2),

where g(β, x_i_) is the dependent variable, β_0_ represents the y intercept, β_1_ is the slope, and $\sigma^{2}$ is the residual error term. This is expressed in a Bayesian network:


(6) [β0,β1,σ2|yi]∝[y|β0,β1,σ2,xi] x [β0|μ0,τ0][β1|μ1,τ1]x [μ0][μ1]x [τ0][τ1] x [σ2],

where *y_i_* represents individual dependent observations, *x_i_* represent the independent observations, and $\mu_{0}\;\mathrm{and}\;\mu_{1}$ are the means and $\tau_{0}\;\mathrm{and}\;\tau_{1}$ represent the standard deviation for the moments of a normal distribution from which $\beta_{0}\;\mathrm{and}{\;\beta }_{1}\;$are drawn. For this example, we will assume that the observations $y_{i}$ and $x_{i}$ are observed perfectly. Readers familiar with the more common expression of a linear regression equation will note the avoidance of the use of ϵ to describe the residual error. This is intentionally done to explicitly demonstrate that the variance arises directly from the probability distribution allowing avoidance of the use of the error term (Hooten and Hefley 2019). A linear regression equation was chosen for two primary reasons, 1) the variables follow a linear relationship with the residuals following a normal distribution, and 2) it is a familiar model commonly used in animal science research. However, if we have reason to believe the equation is nonlinear, we can choose those parameters and their interaction method and fit them by defining them in our Gibbs sampler (rJAGS) code (Figure 6).

**Figure 6:**
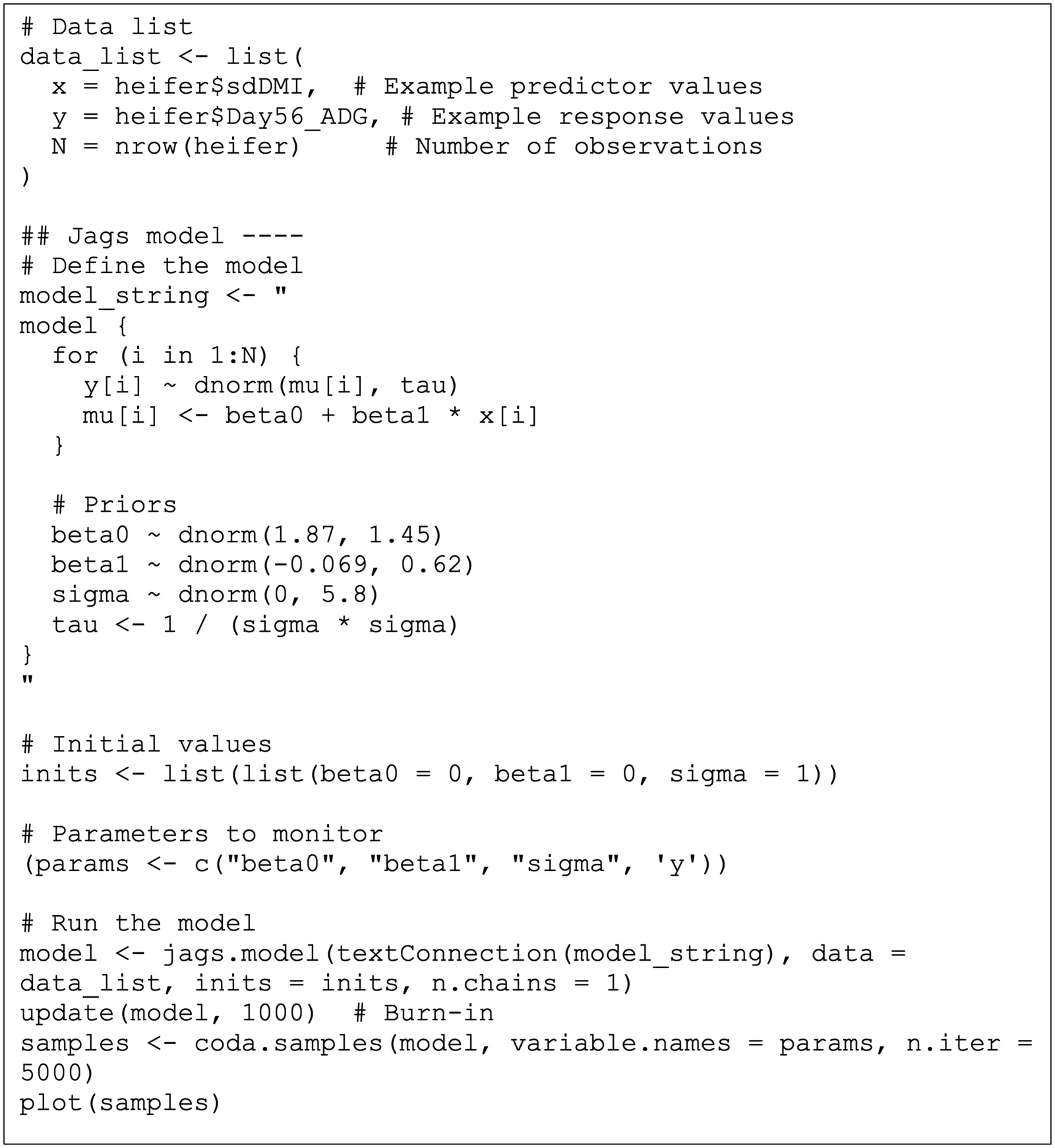
JAGS code with priors used to fit the linear regression model to the data.

#### Defining our priors

To this point, we mentioned above the uniqueness of Bayes theorem for utilizing priors, but we have not yet discussed how exactly priors might be chosen (Bayes theorem). Research here can basically approach this in one of two possible ways. They might choose a flat prior, also known as a vague or uninformative prior, which will reduce its capacity to influence the posterior distributions from our models (Hobbs and Hooten 2015). However, this is not only uninteresting, our final fitted model will return fitted parameter values identical to those calculated using maximum likelihood or frequentist methods, meaning that not only have we done a lot of extra coding for no reason, we also missed the opportunity to interpret our data in mathematically informed by results from previous studies. Thus, a good researcher will carefully choose their priors based upon scientific research. Choosing priors similar to our data also increases the speed of obtaining model convergence in the Markov Chain produced by the Gibbs sampler, but is not completely necessary from a model fitting or computation perspective. Pilot data works well for this, as does a thorough review of the literature for similar projects that report parameters and moments from their statistical distributions. In this case, we turn to a similar study conducted in the lab a few years prior, which conducted a thorough review of animal feeding behavior related to feed efficiency in beef steers (Parsons et al. 2020). Here, it was found β_0_ = 1.87 ± 1.45, β_1_ = −0.069 ± 0.62, and σ is 0 ± 5.80.

Finally, we will use *rjags* and *mcmcVIS* libraries to fit the data to the model and establish the probability density mass functions for each parameter. First, we will define our JAGS model and run the code (Figure 6), and a more thorough explanation is available in the workbook accompanying this manuscript (Parsons 2025).

We allow 1,000 observations to stabilize the model, and the ideal goal is to see the model naturally vary within a given set of values (Figure 7). For more details on Markov Chains and Gibbs samplers, refer to other works such as those referenced here (Gelfand et al. 1990; Hooten and Hefley 2019). Figure 8 illustrates the prior (green), observed (red), and posterior (blue) probability distributions for each of the two parameter values from our fitted model. Note prior probability distribution is much wider, indicating a greater degree of variation and higher uncertainty than the observed data distribution. The posterior distribution, which represents the observed distribution normalized by the prior distribution, is higher than both with a significantly narrower base. This is interpreted as showing agreement between our prior and observed data, which increases the confidence in the posterior distribution for our fitted parameters.

**Figure 7:**
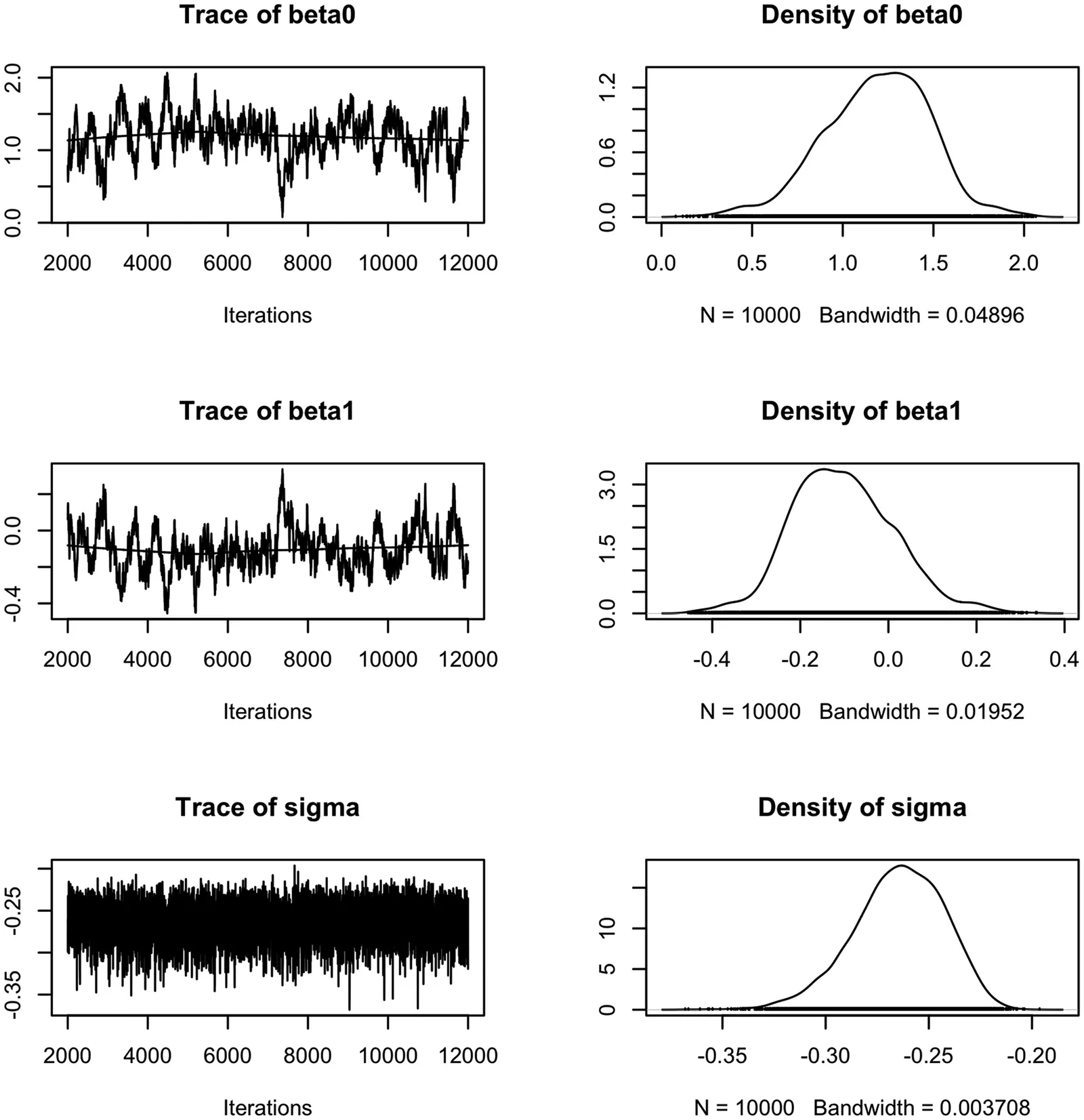
Markov chains and their probability distributions for the three independent parameters of the Bayesian model after removing 1,000 initial runs to allow stabilization.

**Figure 8:**
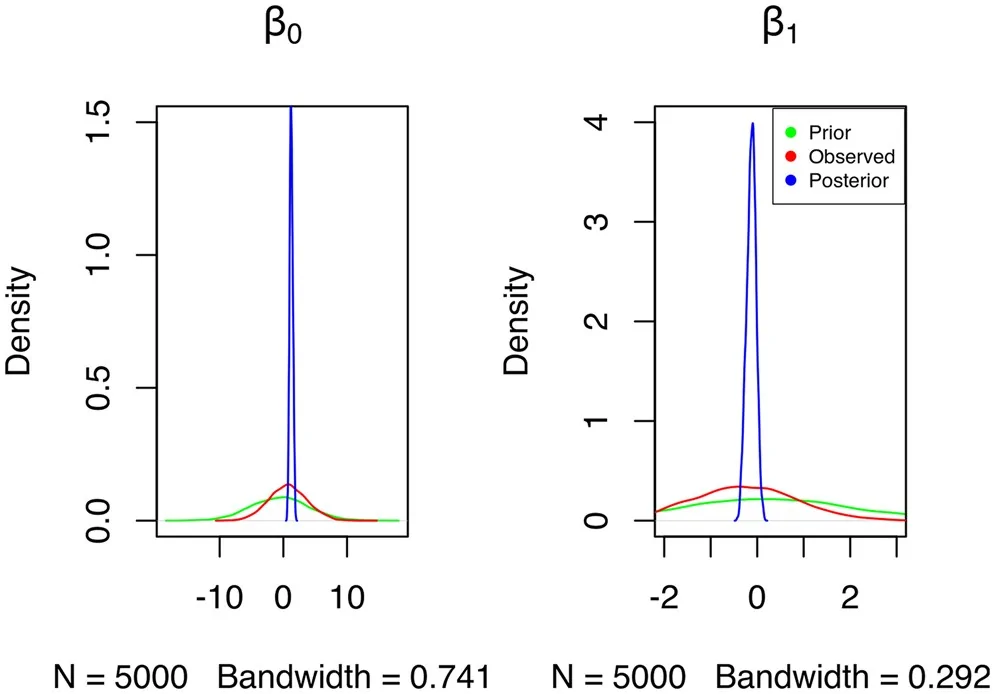
The probability density distributions for the prior (green), observed (red), and posterior (blue) of the parameters fitted from the Bayesian hierarchical model.

#### Case study 1—results

We have successfully developed a research hypothesis, developed a theoretical data model describing the relationship between the variation in feed intake and feed efficiency, identified a specific research objective, and fitted our data model using Bayesian inferential methods. Now, we need to report our findings and provide a decision based upon the available evidence. Making decisions based upon data is known a Statistical Decision Theory, and we assume as scientists that future scientists or livestock managers will seek to weigh our findings in within the context of their operations to make a profitable management decision. Because the data are assumed to present the realization of a random variable *Y* within the context of a probability distribution (ie [y|θ]) all possible outcomes and their probability are presented within support for the outcome of our data. Thus the Bayesian framework allows the final decision maker to make an informed assessment of the data to make decisions which maximize the benefit while minimizing the expected risk (Williams and Hooten 2016).

Figure 9 presents a detailed description of the probable outcomes for each of the fitted β coefficients for the linear regression model. We first note that the mean and standard deviation, indicated by the light-colored vertical bars, are 1.22 ± 0.28 and −0.12 ± 0.11 for β_0_ and β_1_, respectively and the 95% credible interval (the interval encompassing 95% of the potential outcomes) does not encompass zero, indicating a strong effect. As illustrated by the shaded region, β_1_ has a 25% chance of decreasing average daily gain by −0.11 kg/d or more (Figure 9). This represents a substantial potential decrease in both animal welfare and profit. However, now we know in a quantified manner both the magnitude of the potential decrease and the probability of it happening. This represents a major difference from frequentist methods, or at least the way they are applied.

**Figure 9:**
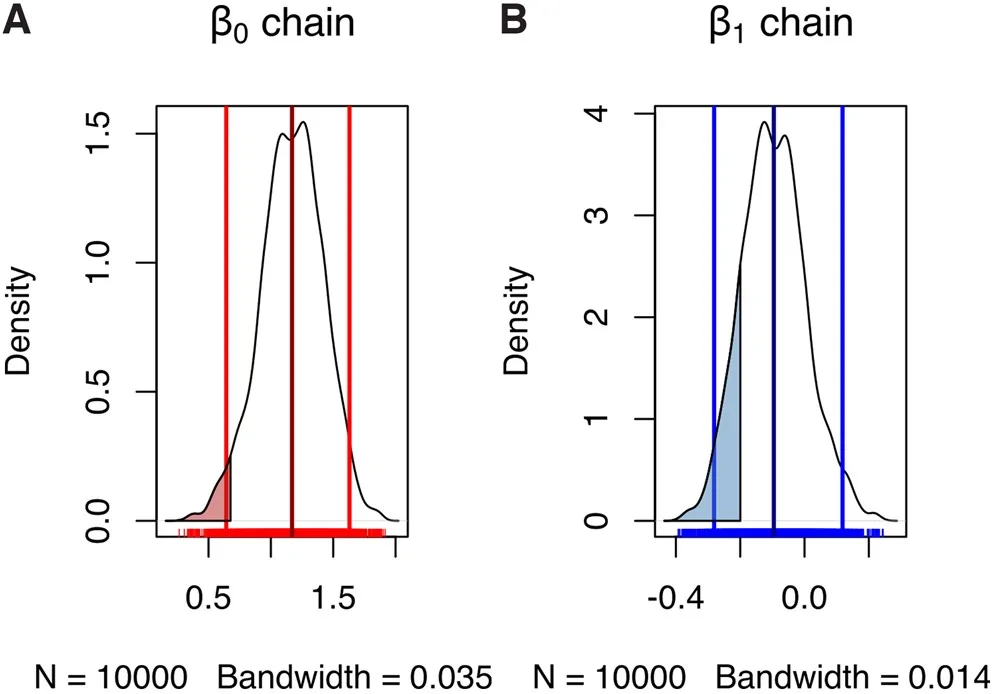
Empirical mass density of the probability distributions of the model parameters β0 (panel A) and β1 (panel B) for the Bayesian model. The mean is denoted by the dark shaded line in the center, with the two outside bars defining the 95% credible interval. The shaded region defines the lowest 25% of observations.

### Case study 2—predicting dry matter intake using biometric output

Dry matter intake is a critical component of beef animal nutrition for ensuring palatability of feedstuffs, ensuring sufficient nutrient intake, and measuring efficiency of growth and performance metrics. It has also been clearly demonstrated that forage quality, as measured by the digestibility of cell wall components as acid detergent fiber, neutral detergent fiber, and overall digestible nutrients are critical for determining both dry matter intake and methane production in grazing animals (Van Soest 1994; National Research Council et al. 2016; McFadden et al. 2025). Precision livestock technologies provide detailed biometric data which can be used to feed nutrition models designed to predict individual animal intake and farther elucidate the relationship between individual biometric variables and overall feed efficiency.

This case study uses data available in the supplementary material of McFadden et al. (2025), and the code and data is available in the work book accompanying this tutorial (Parsons 2025). This study utilized dry angus cows (*n* = 7, BW = 622 ± 11.79 kg) housed at the Cottonwood Field Station fed contrasting forage based diets ad libitum as described by McFadden et al. (2025). Treatments were assigned in a crossover design, with 2 wk acclimation periods allowed between dietary treatments followed by 2 wk data collection periods. During data collection phases, precision technologies were utilized to monitor animal dry matter intake (SmartFeeder), body weight (SmartScale), and CH_4_, O_2_, and CO_2_ production (GreenFeed). The study employed linear regression to facilitate the prediction of dry matter intake using multiple linear regression in the traditional frequentist framework. Here, we will illustrate the application of the Bayesian framework using the same data and modeling approach.

#### Case study 2—objective and model

The objective of this analysis is to “Utilize Bayesian inference to determent the effect of biometric variables such as CH_4_, CO_2_, O_2_ and body weight on dry matter intake prediction equations.” The complete multiple linear regression model used to predict dry matter intake takes on the form of:


(7) g(DMI, xi)=β0+β1CH4+β2CO2+β3O2+β4BW+σ2,

which includes the error notation for the frequentist framework. This converts to a Bayesian Network, which can be expressed probabilistically as:


(8) $$\begin{array}{l} {}[\beta_{0},\beta_{1},\beta_{2},\beta_{3},\beta_{4},p,\sigma^{2}|y_{i}]\propto \\ {}[y_{i}|\beta_{0},\beta_{1},\beta_{2},\beta_{3},\beta_{4},p_{j},x_{i},\sigma^{2}]\;\\ \begin{array}{l} x\;[\beta_{0}|u_{0},t_{0}]\;[\beta_{1}|\mu_{1},\tau_{1}]\;[\beta_{2}|\mu_{2},\tau_{2}][\beta_{3}|\mu_{3},\tau_{3}][\beta_{4}|\mu_{4},\tau_{4}]\;\\ x\;[p_{ij}|\mu_{j}]\\ \begin{array}{l} x\;[\mu_{0}][\mu_{1}][\mu_{2}][\mu_{3}][\mu_{4}]\;\\ \begin{array}{l} x\;[\tau_{0}][\tau_{1}][\tau_{2}][\tau_{3}][\tau_{4}]\\ x\;[\sigma^{2}]. \end{array} \end{array} \end{array} \end{array}$$

With similar symbolism to that described in equation 5, where brackets ([]) indicated a distribution of values, | indicates the value derived from the conjugated distributions of the random variables, β coefficients represent the slope effects of each incremental increase in the independent variable on the dependent variable, μ and τ represent the fixed effects of the moments from a distribution of prior values, and $\sigma^{2}$ is the variation, also known as the residual error, for the fitted equation. You will notice that we have added a variable $p_{ij}$ which takes on the random effect of period and normalizes that across the variables. The resulting Jags code can is seen in Figure 10.

**Figure 10:**
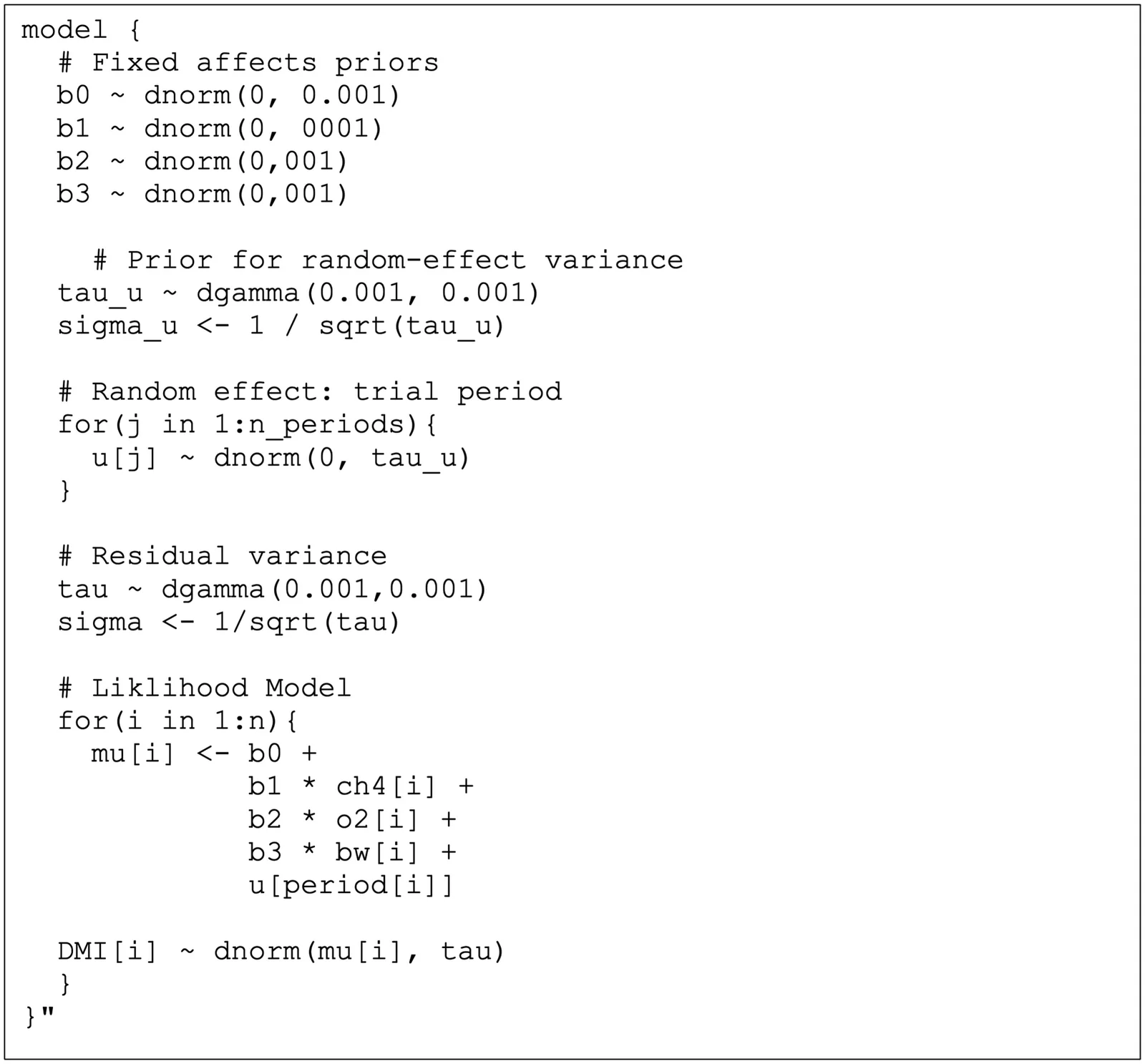
rJags code containing random effect of period for the dry matter intake prediction model.

#### Case study 2—results

Those interested in comparing results may find it useful to further explore the output available in the online workbook available at Parsons (2025), as not all plots are reported here due to space considerations. Examining the results, we first examine the trace plots for the posterior chains to ensure that all chains achieved a stable output (Figure 11), which indicates that we have sufficiently sampled the conjugated distribution to fully form its posterior shape (Hooten and Hefley 2019). This is particularly important for samples with a high degree of variation such as can be seen in the posterior plots for dry matter intake (Figure 12), which is due to the low sample size as noted in McFadden et al. (2025). This necessitates higher resampling efforts resulting in longer Markov Chains to fully sample the posterior chains. However, it also illustrates the ability to utilize smaller datasets in a more efficient manner, while providing a set of priors for use in future research projects.

**Figure 11:**
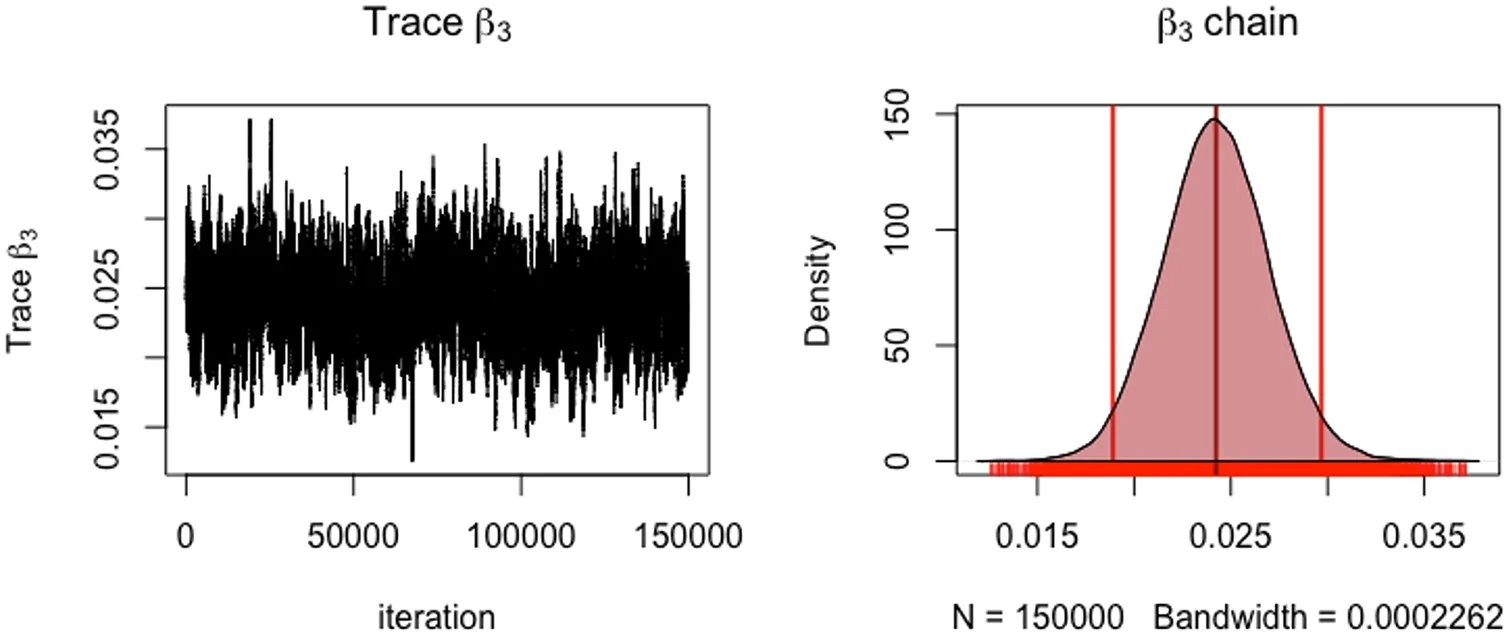
$\beta_{3}$ trace plot and density distribution for bodyweight coefficient on methane production.

**Figure 12:**
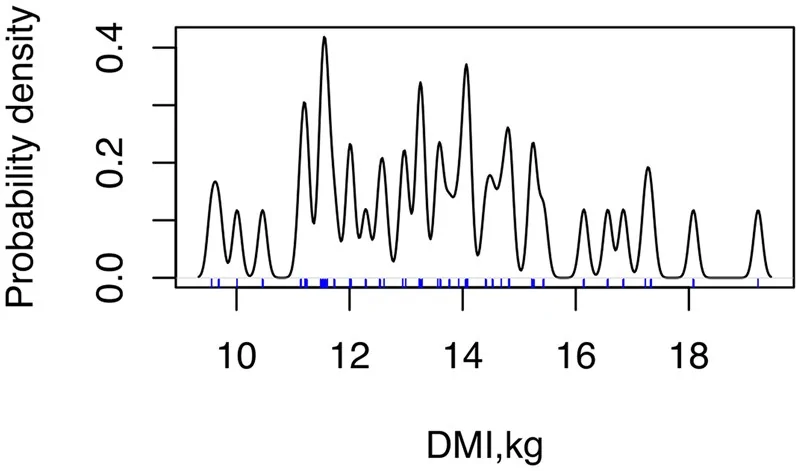
Posterior probability distribution plot for dry matter intake.

The means and 95% credible interval are listed in Table 2. Assessing the 95% credible intervals, we see that both the posterior distributions for the intercept, methane, oxygen, and feeding period contain 0, with relatively small means, indicating that none of these biometric parameters were strong predictors of dry matter intake. The mean for body weight was 0.024, with a credible interval of 0.019–0.03, with over 93% of the density distribution greater than 0. However, it is important to note that even though $\beta_{1}$ (methane) contained zero, 84% of the density interval was greater than 0, indicating that it in combination with body weight were the strongest relationship with dry matter intake. This is in agreement with numerous other studies (National Research Council et al. 2016), and indicates the value of pursuing these two metrics given the shortcomings of the experimental design and equipment failures as noted by the authors in McFadden et al. (2025).

**Table 2:** Means, credible intervals, and probabilities’ posterior distributions of the regression coefficients and random effect for the results from the mixed effects regression model.

Regression parameter	Mean	95% credible interval	Probability ≠ 0
**Intercept,** $\beta_{0}$	−2.77	−6.73–1.47	0.93 < 0
**CH_4_,** $\beta_{1}$	0.004	−0.004–0.013	0.84 > 0
**O_2,_** $\beta_{2}$	0.0	−0.001–0.001	0.42 < 0
**BW,** $\beta_{3}$	0.024	0.019–0.03	0.93 > 0
**Period**	−0.12	−1.54–1.32	0.50 > 0

## Conclusion

Bayesian statistical modeling provides animal scientists with a principled framework for linking biological understanding, prior knowledge, and observed data into a coherent system for inference. By grounding analysis in probability theory and explicitly defining the relationships among variables, Bayesian methods mirror the way biological systems operate—dynamic, uncertain, and informed by accumulated experience. Bayesian methods expand the scientific toolbox by allowing researchers to quantify uncertainty, incorporate prior evidence, and evaluate full distributions of plausible outcomes instead of relying solely on point estimates or binary significance tests.

The case studies presented here illustrate how Bayesian models can be constructed directly from theoretical and empirical understanding of animal behavior, nutrition, and physiology. By defining the process model, specifying appropriate probability distributions, and fitting the model using Markov chain Monte Carlo, researchers gain access to more in depth information about parameter behavior, credible intervals, and the probability of biologically meaningful outcomes. This approach aligns naturally with the scientific process, encouraging researchers to articulate their hypotheses clearly, evaluate evidence transparently, and communicate uncertainty in a way that supports sound decision‑making.

As precision livestock technologies, high‑frequency biometric monitoring, and complex ecological datasets become increasingly common, the need for flexible, transparent, and biologically grounded statistical approaches will only grow. Bayesian inference offers a pathway for meeting that need by allowing scientists to integrate mechanistic understanding with empirical data in a way that is both rigorous and intuitive. By adopting these methods, animal scientists can more effectively design experiments, interpret results, and contribute to a deeper understanding of livestock systems.

## Abbreviations

ADG: average daily gain
BW: body weight
DAG: directed acyclic graph
DMI: dry matter intake
JAGS: just another Gibbs sampler
